# Differences in outcomes after total hip arthroplasty for osteoarthritis between patients with and without central sensitivity syndromes other than fibromyalgia

**DOI:** 10.1038/s41598-022-19369-0

**Published:** 2022-09-12

**Authors:** Yoshihisa Ohashi, Kensuke Fukushima, Kentaro Uchida, Tomohisa Koyama, Maho Tsuchiya, Hiroki Saito, Katsufumi Uchiyama, Naonobu Takahira, Gen Inoue, Masashi Takaso

**Affiliations:** 1 Department of Orthopaedic Surgery, Kitasato University School of Medicine, 1-15-1 Kitasato, Minami-ku, Sagamihara, Kanagawa 252-0374 Japan; 2 Department of Patient Safety and Healthcare Administration, Kitasato University School of Medicine, 1-15-1 Minami-ku, Kitasato, Sagamihara, Kanagawa 252-0374 Japan; 3 Department of Rehabilitation, Kitasato University School of Allied Health Sciences, 1-15-1 Minami-ku, Kitasato, Sagamihara, Kanagawa 252-0374 Japan

**Keywords:** Chronic pain, Pain, Osteoarthritis, Musculoskeletal system

## Abstract

We investigated the differences in outcomes after total hip arthroplasty (THA) for hip osteoarthritis (HOA) between patients with and without central sensitivity syndromes (CSSs) other than fibromyalgia (FM). After excluding two patients with FM, we compared the clinical data of 41 patients with CSSs and 132 patients without CSSs. Clinical data included scores on the central sensitization inventory, visual analog scale for pain (VAS pain), and Japanese Orthopedic Association Hip Disease Evaluation Questionnaire (JHEQ). VAS pain was significantly higher at 3 and 6 months after THA in patients with CSSs than in those without CSSs (3 and 6 months, P < 0.001). Satisfaction, pain, and mental JHEQ scores were lower in patients with CSSs than in those without CSSs (satisfaction, P < 0.001; pain, P = 0.011; mental, P = 0.032). Multiple regression analyses indicated that one and ≥ 2 CSS diagnoses significantly impacted the satisfaction score (one CSS, β = − 0.181, P = 0.019; ≥ 2 CSSs, β = − 0.175, P = 0.023). Two or more CSSs were the only factor influencing the pain score (β = − 0.175, P = 0.027). Pain in patients with CSSs reflects central sensitization, which may adversely affect post-operative outcomes. Surgeons should pay attention to patients with a history of CSSs diagnoses who undergo THA for HOA.

## Introduction

Total hip arthroplasty (THA) is an effective surgical intervention for patients with persistent painful hip osteoarthritis (HOA), improving not only pain relief, but also the quality of life, gait function, cardiorespiratory function, and patient satisfaction^1–3^. Demand for the THA procedure is expected to continue to increase worldwide^4,5^. However, evidence indicates that despite the absence of post-operative structural complications, approximately 10% of patients with HOA who undergo primary THA report residual pain and dissatisfaction each^6,7^. The causes of these post-operative adverse outcomes are not well understood.

In the past decade, central sensitization (CS), defined as increased responsiveness of nociceptive neurons in the central nervous system to normal or subthreshold afferent input, has been suggested to contribute to the persistence and extent of pain in HOA and knee osteoarthritis (KOA)^8,9^. A high degree of pre-operative CS has been reported as a risk factor for residual pain at 1-year post-operatively in patients who undergo THA for HOA^10^. Furthermore, both pre-operatively and post-operatively, CS is resistant to treatment, including traditional physiotherapy and pain medication; however, specific treatments have been found to be effective^11,12^. Therefore, identifying the characteristics of patients with HOA with CS components is an important endeavor, as it may influence the selection of treatment for HOA.

Central sensitivity syndrome (CSS) is a generic term for a group of disorders in which CS may be the root etiology^13^. A lowered threshold for the perception of sensory information, which is a common condition in these disorders, is closely linked to the exacerbation and persistence of pain^14^. A previous study has reported that approximately 27% of patients with musculoskeletal disorders have at least one CSS^15^. Furthermore, some studies have described poor post-operative outcomes in patients with musculoskeletal disorders and fibromyalgia (FM), a typical CSS^16,17^. Although other CSSs have a similar pathophysiology to FM, post-operative outcomes in patients with CSSs other than FM who undergo THA for HOA remain unclear.

Therefore, we investigated the pre-operative clinical characteristics and post-operative outcomes after THA for HOA, including pain and satisfaction, in patients with and without CSSs, excluding FM.

## Methods and materials

### Participants

**Figure 1:**
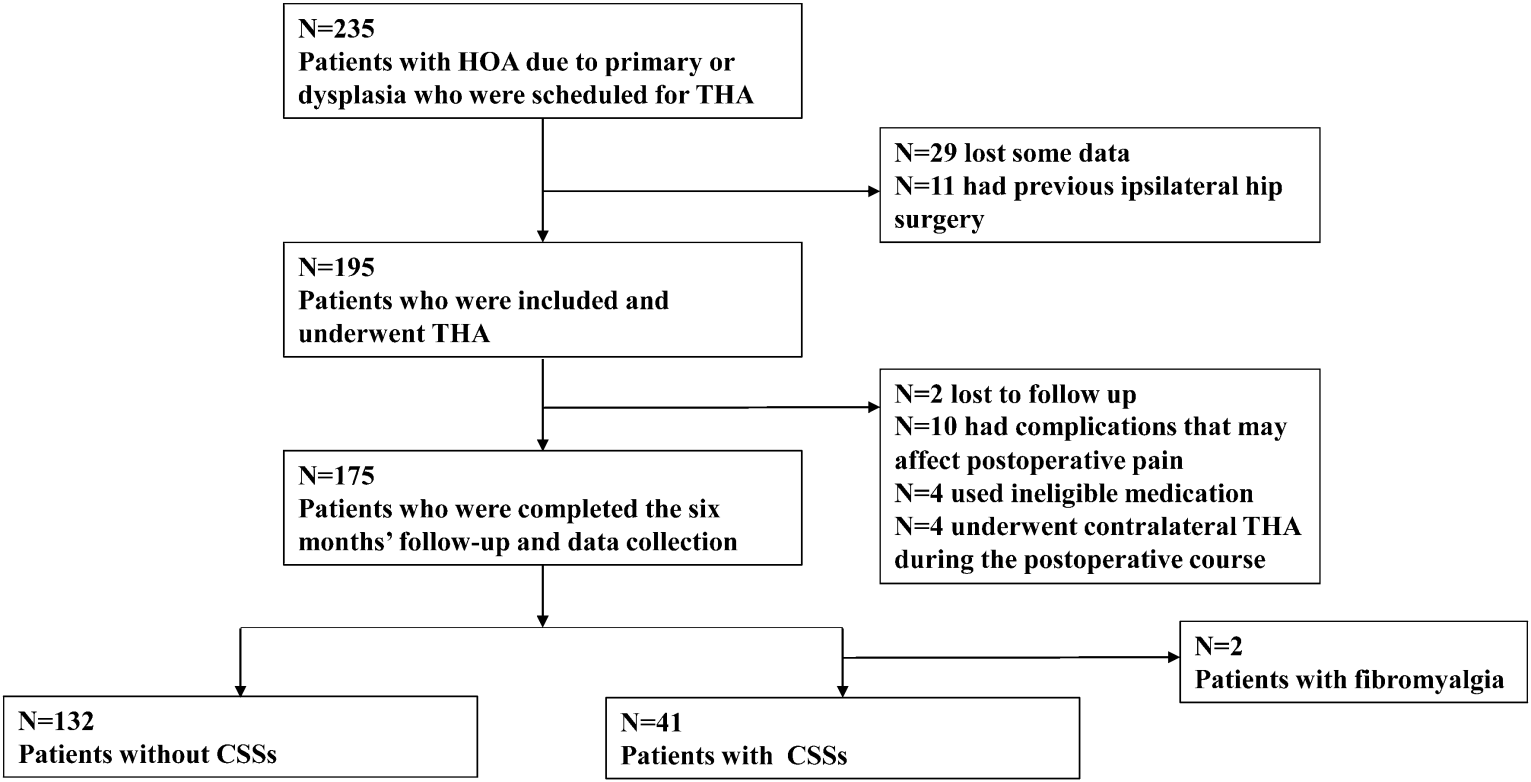
Flowchart of the study. *HOA* hip osteoarthritis, *THA* total hip arthroplasty, *CSS* central sensitivity syndrome.

This retrospective study was approved by the institutional review board of our institution. All patients provided informed consent and the study was conducted in accordance with the Declaration of Helsinki. We retrospectively enrolled 235 consecutive patients with HOA due to primary or developmental dysplasia of the hip who underwent THA at a single center between January 2019 and March 2021. All THA procedures were performed under general anesthesia using a minimally invasive anterolateral supine approach. Cementless type implants of both cups and stems were used in all cases. A fully hydroxyapatite-coated stem was mainly used; Zweymüller and modular Wagner-cone types were also used according to the shape of the patient’s medullary cavity. Of the enrolled patients, 29 with missing data and 11 with a previous history of any hip surgery on the same laterality were excluded. Further, ten patients with any peri- or post-operative complications that affect post-operative pain (infection, peri-operative fractures, and implant loosening); four patients who used centrally acting agents, opioids, and/or steroids; patients who underwent contralateral THA during the post-operative course in this study period; and two patients who did not complete the 6-month follow-up after THA were excluded. Finally, 175 patients were included in this study (Fig. 1).

### Identification of patients with central sensitivity syndromes

The CS inventory (CSI) is a screening tool for identifying the presence of CSSs^15,18^. The CSI consists of parts A and B. Part A comprises a scoring system with 25 self-reported items on symptoms associated with CS, scored from 0 to 100 points, with 0 and 100 being the best and worst scores, respectively. Part B can be used to screen for previous diagnoses of CSSs, including restless legs syndrome, chronic fatigue syndrome, FM, temporomandibular joint disorder, migraine or tension headaches, irritable bowel syndrome, multiple chemical sensitivities, neck injuries, anxiety or panic attacks, and depression. These disorders have been shown to be related to CS components and are included in the CSS family^13,14^. CSI parts A and B were evaluated 1 month before surgery. The number (percentage) of patients with specific CSSs was calculated using CSI part B. After excluding patients with FM, we divided the patients into two groups: the patients with one or more CSSs and those without CSSs. In addition, the number of CSSs diagnoses was determined for each patient and patients with CSSs were categorized as having one CSS or ≥ 2 CSSs.

### Assessment of clinical data

Pain was evaluated using the visual analog scale for pain (VAS pain: 0 mm, no pain; 100 mm, worst possible pain). Post-operative outcomes were evaluated using the scoring system of the Japanese Orthopedic Association Hip-Disease Evaluation Questionnaire (JHEQ)^19^. JHEQ has been widely used as a patient-reported outcome measure in Japan to assess the condition of the hip joint. JHEQ consists of items on pain (0–28 points), function (0–28 points), and mental (0–28 points), with 0 and 28 representing the worst and best scores, respectively. Additionally, satisfaction was evaluated using the VAS for satisfaction (VAS satisfaction: 0 mm, most dissatisfaction; 100 mm, most satisfaction) included in JHEQ. VAS pain was evaluated 1 month before, and 3 and 6 months post-operatively, and JHEQ scores were evaluated at 6 months post-operatively. HOA was assessed using the Kellgren–Lawrence (KL) classification system^20^. All patients were categorized as having an advanced or end-stage disease (KL grade 3 or 4). Furthermore, we investigated the correlations between the pre-operative CSI (part A) score and post-operative VAS pain and JHEQ scores in patients with and without CSSs. The differences in post-operative outcomes evaluated by VAS pain and JHEQ scores between the patients with and without CSSs were also investigated.

### Statistical analysis

Results are expressed as the mean ± standard error of the mean unless otherwise indicated. In order to eliminate the bias of influences of FM on post-operative outcomes, two patients with FM were excluded from comparisons in pre- and post-operative clinical data between patients with and without CSSs. Age, height, weight, body mass index (BMI), duration of hip pain, CSI score, VAS pain, and JHEQ scores were analyzed as continuous variables; sex, HOA grade (as assessed by the KL classification system), and CSS diagnoses were analyzed as nominal scale variables. Continuous and categorical variables were compared between groups using the Mann–Whitney *U* test and Pearson's Chi-square test, respectively. The correlations between the pre-operative CSI score and post-operative VAS pain and JHEQ scores in patients with and without CSSs were analyzed using the Spearman’s correlation coefficient. Correlation coefficient was represented by ρ. The effect size of differences in VAS pain and JHEQ scores between the two groups was assessed using Cohen’s d. Multivariate regression analysis was used to identify differences in post-operative JHEQ scores according to the number of CSS diagnoses (one or ≥ 2). All models were adjusted for sex, age, BMI, duration of hip pain, and VAS pain at 1 month pre-operatively. Statistical significance was set at P < 0.05. Statistical analyses were performed using SPSS for Windows (version 28.0; IBM Corp., Armonk, NY, USA).

## Results

### Prevalence of central sensitivity syndromes in patients with hip osteoarthritis before total hip arthroplasty

The number (prevalence) of patients with CSSs is shown in Table 1. Migraine or tension headaches; neck injury, including whiplash; depression; temporomandibular joint disorder; irritable bowel syndrome; anxiety or panic attacks; and FM were present in 8.0%, 7.4%, 5.7%, 5.1%, 1.7%, 1.1%, and 1.1% of patients, respectively. Additionally, 0.6% of patients had a history of restless leg syndrome, multiple chemical sensitivities, and chronic fatigue syndrome. Furthermore, 18.3%, 4.6%, 1.1%, and 0.6% of patients were diagnosed with 1–4 CSSs, respectively. After excluding patients with FM, 41 patients with one or more CSSs and 132 patients without CSSs were analyzed (Fig. 1). Demographic and clinical factors of patients with and without CSSs are shown in Table 2. The proportion of women was higher among patients with CSSs than among those without CSSs (P = 0.010). The CSI score was higher in patients with CSSs than in patients without CSSs (P < 0.001). There were no significant differences in other factors between the two groups.

**Table 1:** Central sensitivity syndrome prevalence in patients with hip osteoarthritis (N = 175).

	N (%)
**Number of patients with each CSS**	
Migraine or tension headaches	14 (8.0)
Neck injury, including whiplash	13 (7.4)
Depression	10 (5.7)
Temporomandibular joint disorder	9 (5.1)
Irritable bowel syndrome	3 (1.7)
Anxiety or panic attacks	2 (1.1)
Fibromyalgia	2 (1.1)
Restless leg syndrome	1 (0.6)
Multiple chemical sensitivities	1 (0.6)
Chronic fatigue syndrome	1 (0.6)
**Number of patients with one CSS**	32 (18.3)
**Number of patients with two CSSs**	8 (4.6)
**Number of patients with three CSSs**	2 (1.1)
**Number of patients with four CSSs**	1 (0.6)

*CSS* central sensitivity syndrome.

**Table 2:** Demographic and clinical factors.

	Patients without CSSs N = 132	Patients with CSSs N = 41	P-values
Sex, male/female, N	25/107	1/40	**0.010**
Age (years)	64.9 ± 1.0	63.8 ± 1.9	0.819
Height (cm)	156.1 ± 0.7	155.2 ± 0.9	0.959
Weight (kg)	60.3 ± 1.3	59.0 ± 2.0	0.996
Body mass index (kg/m^2^)	24.6 ± 0.4	24.4 ± 0.7	0.828
Hip osteoarthritis grade (KL 3/4), N	48/84	15/26	0.875
Duration of hip pain (months)	51.7 ± 4.3	44.4 ± 6.6	0.408
CSI score	18.5 ± 0.9	28.0 ± 1.8	**< 0.001**

The results are expressed as the mean ± standard error of the mean, unless otherwise indicated.

Statistically significant P-values (< 0.05) are indicated in bold.

*CSS* central sensitivity syndrome, *KL* Kellgren–Lawrence, *CSI* central sensitization inventory.

### Correlations between the pre-operative central sensitization inventory score and post-operative clinical outcomes in patients with and without central sensitivity syndromes

Table 3 indicates the correlations between the pre-operative CSI score and post-operative JHEQ and VAS pain scores in patients with and without CSSs. The CSI score correlated negatively with the pain, function, mental, and total scores on JHEQ in patients without CSSs (pain, ρ = − 0.291, P < 0.001; function, ρ = − 0.209, P = 0.016; mental, ρ = − 0.334, P < 0.001; total, ρ = − 0.305, P < 0.001). In contrast, there were no significant correlations between the CSI score and all clinical outcomes in patients with CSSs.

**Table 3:** Correlations between the pre-operative central sensitization inventory score and post-operative clinical outcomes in patients with and without central sensitivity syndromes.

	VAS pain	VAS satisfaction	Pain score	Function score	Mental score	Total score
Patients with CSSs (N = 41)	ρ = 0.201	ρ = − 0.204	ρ = − 0.097	ρ = − 0.116	ρ = − 0.301	ρ = − 0.224
	P = 0.213	P = 0.206	P = 0.550	P = 0.450	P = 0.059	P = 0.164
Patients without CSSs (N = 132)	ρ = 0.081	ρ = 0.039	ρ = − 0.291	ρ = − 0.209	ρ = − 0.334	ρ = − 0.305
	P = 0.353	P = 0.654	P < 0.001^‡^	P = 0.016*	P < 0.001^‡^	P < 0.001^‡^

VAS satisfaction, pain, function, mental, and total scores were examined using the Japanese Orthopaedic Association Hip-Disease Evaluation Questionnaire. Correlation coefficient is represented by ρ. P < 0.05 indicates statistical significance. *P < 0.05 and ^‡^P < 0.001.

*CSI* central sensitization inventory, *VAS* visual analog scale, *CSS* central sensitivity syndrome.

### Changes in visual analog scale pain score before and after total hip arthroplasty in patients with and without central sensitivity syndromes

**Figure 2:**
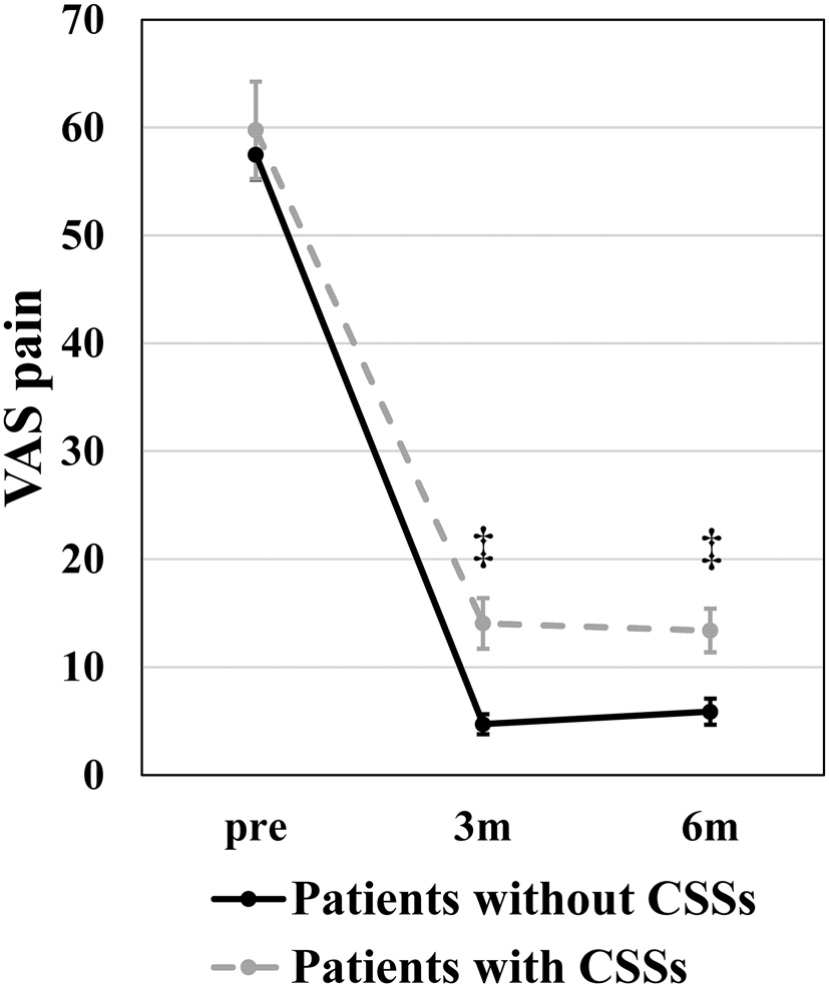
VAS pain scores at 1 month before, and 3 and 6 months after total hip arthroplasty in patients with and without CSSs. ^‡^P < 0.001. *VAS* visual analog scale, *CSS* central sensitivity syndrome, *Pre* 1 month before surgery, *3m* 3 months after surgery, *6m* 6 months after surgery.

Figure 2 shows the VAS pain scores at 1 month pre-operatively and at 3 and 6 months post-operatively in patients with and without CSSs. At baseline (1 month pre-operatively), VAS pain scores did not significantly differ between the two groups (P = 0.456, Cohen’s d = 0.100). However, VAS pain scores were significantly higher at 3 and 6 months post-operatively in patients with CSSs than in those without CSSs (3 months, P < 0.001, Cohen’s d = 0.788; 6 months, P < 0.001, Cohen’s d = 0.556).

### Differences in Japanese Orthopedic Association Hip Disease Evaluation Questionnaire scores after total hip arthroplasty in patients with and without central sensitivity syndromes

**Figure 3:**
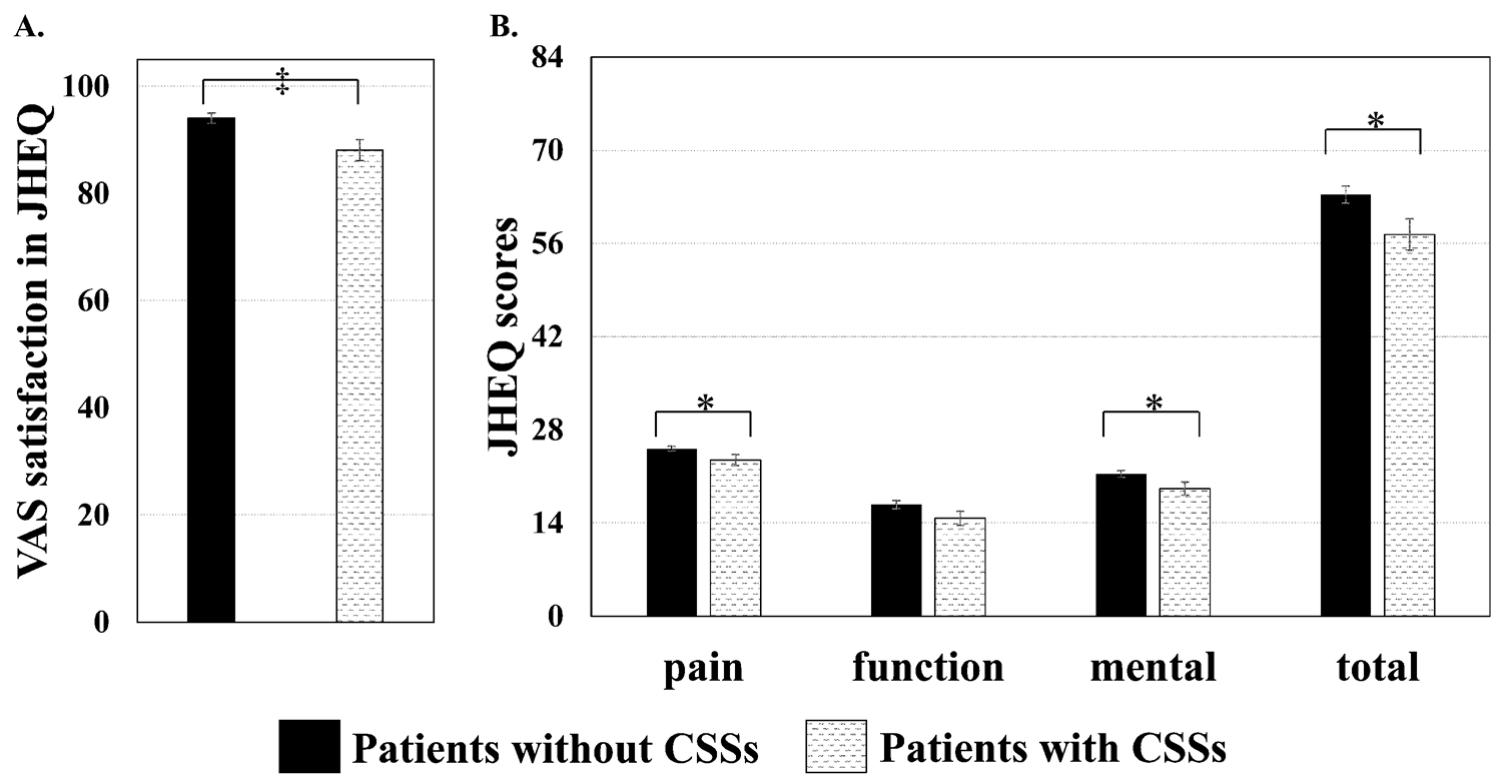
JHEQ scores at 6 months after total hip arthroplasty in patients with and without CSSs. (**A**) VAS satisfaction on JHEQ. (**B**) Pain, function, mental, and total scores on JHEQ. *P < 0.05 and ^‡^P < 0.001. *VAS* visual analog scale, *CSS* central sensitivity syndrome, *JHEQ* Japanese Orthopedic Association Hip-Disease Evaluation Questionnaire.

Figure 3 indicates differences in JHEQ scores at 6 months after THA between the two groups. VAS satisfaction on JHEQ was lower in patients with CSSs than in those without CSSs (P < 0.001, Cohen’s d = 0.538). Pain, mental, and total scores on JHEQ were also lower in patients with CSSs than in those without CSSs (pain, P = 0.011, Cohen’s d = 0.400; mental, P = 0.032, Cohen’s d = 0.343; total, P = 0.026, Cohen’s d = 0.393). The functional score did not significantly differ between the two groups (P = 0.174, Cohen’s d = 0.271).

### Differences in Japanese Orthopedic Association Hip Disease Evaluation Questionnaire scores after total hip arthroplast according to the number of central sensitivity syndromes

Table 4 shows the influence of the number of CSSs diagnoses (one or ≥ 2) on JHEQ scores after THA in patients with HOA. Both one and ≥ 2 CSS diagnoses impacted VAS satisfaction on JHEQ (one CSS, β = − 0.181, P = 0.019; ≥ 2 CSSs, β = − 0.175, P = 0.023). Furthermore, the presence of ≥ 2 CSSs was the only factor that significantly impacted the pain score on JHEQ (one CSS, β = − 0.093, P = 0.233; ≥ 2 CSSs, β = − 0.175, P = 0.027). Borderline significance (0.05 < P < 0.18) was found between one CSS and mental score, both one and ≥ 2 CSS diagnoses and total scores (mental score, one CSS, β = − 0.110, P = 0.163, ≥ 2 CSSs, β = − 0.073, P = 0.356; total score, one CSS, β = − 0.105, P = 0.177, ≥ 2 CSSs, β = − 0.122, P = 0.142).

**Table 4:** Impact of the number of central sensitivity syndrome diagnoses on post-operative Japanese Orthopaedic Association Hip-Disease Evaluation Questionnaire scores in patients with hip osteoarthritis: multiple regression analyses (N = 173).

Outcome (JHEQ)	The number of CSSs diagnoses	β	95% Confidence interval	P-values	R^2^ (adjusted R^2^)
Satisfaction	One	− 0.181	− 0.331 to − 0.031	**0.019**	0.105 (0.067)
	Two or more	− 0.175	− 0.327 to − 0.025	**0.023**	
Pain	One	− 0.093	− 0.247 to 0.060	0.233	0.062 (0.022)
	Two or more	− 0.175	− 0.329 to − 0.020	**0.027**	
Function	One	− 0.067	− 0.221 to 0.089	0.399	0.051 (0.010)
	Two or more	− 0.067	− 0.222 to 0.088	0.397	
Mental	One	− 0.110	− 0.265 to 0.045	0.163	0.051 (0.010)
	Two or more	− 0.073	− 0.228 to 0.082	0.356	
Total	One	− 0.105	− 0.259 to 0.048	0.177	0.067 (0.027)
	Two or more	− 0.122	− 0.278 to 0.035	0.142	

All models were adjusted for sex, age, body mass index, duration of hip pain, and pre-operative visual analog scale for pain. Statistically significant P-values (< 0.05) are indicated in bold.

*CSS* central sensitivity syndrome, *JHEQ* Japanese Orthopaedic Association Hip-Disease Evaluation Questionnaire, *β* standardized partial regression coefficient.

## Discussion

In the present study, approximately one-quarter of the patients who underwent THA for HOA had at least one CSS, which is consistent with several previous reports on the prevalence of CSSs in patients with musculoskeletal diseases^15,21,22^. However, the present study is the first to directly indicate poorer post-operative outcomes, including satisfaction, pain, and mental scores, in patients with CSSs other than FM who underwent THA for HOA than in those without CSSs. These results are important because they indicate that CSSs are one of the factors influencing pain persistence after THA.

We also investigated the correlations between pre-operative CSI scores and post-operative clinical outcomes in HOA patients with and without CSSs. Several studies indicated the associations between pre-operative CSI scores and poor post-operative outcomes in patients with HOA and KOA^10,23,24^. As in previous studies, the CSI score correlated with pain, function, mental, and total scores on the JHEQ in patients with HOA without CSSs in this study. In contrast, we did not find significant correlations between the CSI score and all clinical outcomes in patients with CSSs. Patients with CSSs may influence post-operative outcomes regardless of CSI score.

In our result, more persistent pain was observed in patients with CSSs than in those without CSSs at 3 and 6 months after THA. Additionally, patients with CSSs had significantly lower pain scores on JHEQ at 6 months post-operatively than those without CSSs. Previous reports have suggested that CSSs are associated with a lower pain threshold, which is related to CS^14^. Furthermore, Brummett et al. reported that patients with higher scores on an FM survey, which assessed widespread pain and comorbid symptoms, had poor outcomes 6 months after primary total hip and knee arthroplasty^16^. Other CSSs may also affect persistent post-operative pain because they have pathophysiological processes similar to FM^13,14^.

CS in osteoarthritis is triggered by an increased expression of various cytokines, including nerve growth factor (NGF), in synovial inflammation^25^. Furthermore, NGF gene expression in the synovium was reported as correlated with pre-operative CSI and pain scores in 50 patients with HOA^26^. Taken together, the present results and those of previous studies suggest that comorbidities of the pathophysiology of HOA and CSS may be a cause of persistent post-operative pain.

In addition to a pain score, JHEQ provides post-operative satisfaction, mental, and functional scores in patients with HOA^19,27^. In the present study, satisfaction and mental scores were also significantly lower in patients with CSSs than in those without CSSs at 6 months after THA. Several factors are closely related to post-operative dissatisfaction^23,28–30^. Baker et al. indicated that persistent pain after total knee arthroplasty (TKA) was the strongest predictor of low satisfaction^28^. Kim et al. demonstrated that patients with KOA with CS (CSI score ≥ 40) reported severe pain, which was correlated with the satisfaction score, at 3 months after TKA^23^. Several studies have also suggested that poor mental health status and depression prior to THA or TKA are associated with increased post-operative dissatisfaction scores^29,30^. Thus, suffering from CSSs may exacerbate post-operative pain and psychological disorders, affecting satisfaction.

The present study also investigated the impact of the number of CSS diagnoses on post-operative JHEQ scores in patients who underwent THA for HOA using multiple regression analyses. Both one and ≥ 2 CSSs diagnoses impacted the satisfaction score. The presence of ≥ 2 CSSs was the only significant factor influencing the pain score on JHEQ. Further, there was near-marginal significance (0.05 < P < 0.18) between one CSS and mental score, both one and ≥ 2 CSSs and total scores on JHEQ, respectively. The relationship between poor post-operative outcomes and the number of CSSs is still controversial. Several studies have reported a correlation between the pre-operative degree of CS and poor outcomes, including persistent pain after THA^10,31^. However, to our best knowledge, no study has previously indicated differences in post-operative outcomes according to the number of CSSs morbidities. These previous studies and our results showed that increased CSS morbidities might exacerbate CS and affect persistent post-operative pain and dissatisfaction.

The current study had several limitations. First, the number of patients with CSSs in the current study was relatively small to analyze in the regression analysis. In addition, we could not assess the relationships between the poor post-operative outcomes and each CSS due to the small sample size. Further investigations will be needed. Second, we used only JHEQ to evaluate post-operative outcomes 6 months after THA in this study. JHEQ has been a widely used patient-reported outcome measure in Japan because it includes specific items related to the Japanese lifestyle. Seki et al. reported the reliability and validity of JHEQ to compare the SF-36 questionnaire as a generic quality of life scale and the Oxford hip score as a disease-specific scale used worldwide^27^. We suspect that JHEQ may be a useful tool in the evaluation of hip joint clinical conditions even in other ethnic patients, but other scales used worldwide might be needed to evaluate together. Third, the assessment of CSS diagnoses was not performed independently at our institution and comprised merely an aggregation of previous diagnoses made at other institutions. Therefore, the present study did not consider the medical conditions and medications for CSSs in individual cases. Despite these limitations, we believe that the present study provides valuable information on the importance of considering CSSs in patients who undergo THA for HOA, as they may be at risk for poor post-operative outcomes.

## Conclusions

The present results indicate poorer post-operative outcomes, including persistent pain and dissatisfaction, after THA for HOA in patients with CSSs, excluding FM, than in those without CSSs. Pain in patients with CSSs reflects CS, which may adversely affect post-operative outcomes. Surgeons should pay close attention to patients who undergo THA for HOA with CSSs, even those without FM.

## Data Availability

The datasets supporting the conclusions of this article are included within the article. The raw data can be requested from the corresponding author, KF.
